# Rising Threats of MRSA and Carbapenem-Resistant *Acinetobacter* in Residential Care Homes for the Elderly During COVID-19 in Hong Kong

**DOI:** 10.3390/microorganisms13081912

**Published:** 2025-08-16

**Authors:** Edmond Siu-Keung Ma, Shuk-Ching Wong, Vincent Chi-Chung Cheng, Enoch Hsu, Hong Chen, Edwin Lok-Kin Tsui

**Affiliations:** 1 Infection Control Branch, Centre for Health Protection, Department of Health, Hong Kong SAR, China; enoch_hsu@dh.gov.hk (E.H.);; 2 Infection Control Team, Queen Mary Hospital, Hong Kong West Cluster, Hong Kong SAR, Chinavcccheng@hku.hk (V.C.-C.C.); 3 School of Nursing, Li Ka Shing Faculty of Medicine, The University of Hong Kong, Pokfulam, Hong Kong SAR, China; 4 Department of Microbiology, School of Clinical Medicine, Li Ka Shing Faculty of Medicine, The University of Hong Kong, Pokfulam, Hong Kong SAR, China; 5 Department of Microbiology, Queen Mary Hospital, Hong Kong SAR, China; 6 Centre for Health Protection, Department of Health, Hong Kong SAR, China

**Keywords:** antimicrobial resistance, methicillin-resistant *Staphylococcus aureus*, carbapenem-resistant *Acinetobacter*, epidemiology, infection control

## Abstract

Methicillin-resistant *Staphylococcus aureus* (MRSA) and carbapenem-resistant *Acinetobacter* (CRA) cause significant mortality and morbidity among the elderly population. We conducted a territory-wide point prevalence survey in Hong Kong to estimate the prevalence of MRSA and resistant *Acinetobacter* among residents of residential care homes of the elderly (RCHEs). A total of 26 RCHEs with 1529 residents were recruited, including 20 private homes and 6 non-private homes. The size of the homes ranged from 13 to 135 residents, with a median of 57 residents. Overall, the prevalence rates of MRSA, CRA, and multidrug-resistant *Acinetobacter* were 33.9% (95% CI: 31.5–36.3%), 8.1% (95% CI: 6.8–9.6%), and 0.8% (95% CI: 0.4–1.4%), respectively. Private homes had a greater prevalence of MDROs than non-private homes did, whereas RCHEs in the Hong Kong region had a greater prevalence of most resistant organisms, followed by those in the Kowloon region and then those in the New Territories. We detected a high prevalence of MRSA during the COVID-19 pandemic, with additional information on CRA that was not previously known. Continuous surveillance and stringent infection control measures are needed to combat these resistant pathogens among this vulnerable population.

## 1. Background

A global disease burden study on antimicrobial resistance has estimated that, in 2021, there were 4.71 million deaths associated with bacterial AMR, including 1.14 million deaths attributable to bacterial AMR [1]. Among all drug-resistant bacteria, *Staphylococcus aureus* and *Acinetobacter baumannii* are the first and second most common pathogens, accounting for 18.8% and 15.2% of AMR-attributable deaths among patients aged 5 years and older, respectively [1]. According to the 2022 Global Antimicrobial Resistance and Use Surveillance System (GLASS) report published by the World Health Organisation (WHO), the median reported rate for methicillin-resistant *Staphylococcus aureus* (MRSA) is 34.7%, and the resistance rate to carbapenems for Acinetobacter spp. is 69.0 to 73.4%, depending on the individual antibiotics [2]. The situation of AMR has worsened during the coronavirus disease 2019 (COVID-19) pandemic, as evidenced by a systematic review of 30 studies that revealed increased rates of MRSA and carbapenem-resistant *Acinetobacter baumannii* (CRAB) during this period [3]. In Hong Kong, although there was no significant increase in the trend of MRSA and ESBL-producing Enterobacterales infections, a significant increase in the trend of carbapenem-resistant *Acinetobacter* (CRA) infections was observed in one healthcare region [4]. Combating AMR during the COVID-19 pandemic was a critical concern [5,6]. In 2024, the WHO published a list of bacterial priority pathogens to guide research, development, and strategies to prevent and control AMR, and CRAB and MRSA are classified under the critical and high-risk groups in the priority list, indicating the importance of their control [7].

The WHO estimates that AMR-related infections in Hong Kong from 2020 to 2030 will result in 18,433 excess deaths and incur a total economic cost of USD 4.3 billion [8]. In a local study, the annual incidence rates of MRSA in individuals aged 20–59 years and ≥60 years were 0.96–1.148 per 100,000 and 22.7–24.8 per 100,000, respectively [9]. The 30-day mortality rate was 367 (32.39%), and older patients (>79 years), chronic lung disease, and prior hospitalisation were associated with increased mortality [9]. For CRAB, we have previously detected diverse multilocus sequence typing among RCHE residents (6.5%) with a high background rate of MRSA (32.2%) [10]. In addition, residential care homes for the elderly (RCHEs) are considered high-risk areas for the acquisition of multidrug-resistant organisms (MDROs), which are known to be more common among patients with chronic illness, who are immunocompromised or dependent, who have wounds, and who are frequently hospitalised [11,12]. Previous prevalence studies have revealed an increasing trend of MRSA among RCHEs in Hong Kong, increasing from 5.1% (80/1563) in 2005 to 21.6% (436/2020) in 2011 [13,14]. The prevalence of MRSA subsequently increased to 30.1% (282/1207) in 2015 [15]. In 2021, we collected samples from 1273 RCHE residents in one of the regions in Hong Kong and reported that 48.7% carried MRSA at any site, whereas 8.5% of staff members were nasal MRSA carriers, predominantly of the ST 1047 lineage 1 [16]. The COVID-19 pandemic has worsened AMR by disrupting hospital infection control practices, resulting in more secondary bacterial infections and increasing the utilisation of antibiotics for inpatients [3,5]. MRSA and CRA have been identified as the key pathogens for implementing aggressive infection control measures in RCHE, as mentioned in the Hong Kong Strategy and Action Plan on AMR [17]. It is therefore worth tracking the latest trends of MRSA and CRA, particularly when COVID-19 activity is high, which might compromise infection control practices in RCHEs.

## 2. Methods

### 2.1. Recruitment and Sampling of RCHE Residents

We conducted a point prevalence survey to estimate the prevalence of MRSA and resistant *Acinetobacter* among residents of RCHEs. Stratified single-stage cluster sampling was conducted on the basis of the RCHE type (private or non-private), geographical location, and RCHE size (≤100 residents, >100 residents) to ensure the representativeness of these key factors, which may affect the prevalence of MDROs. We obtained the list of RCHEs from the Social Welfare Department. Residents were included in the survey if they were present in the RCHEs on the survey day; those who were discharged, admitted to the hospital, or on home leave on that day were not included. Additionally, residents had to agree to specimen collection when approached by nursing staff.

The sample size was calculated on the basis of the estimated MRSA incidence, which was the primary objective of this study, using the following equation:

$$N=1.96^{2}\frac{\left(1-prevalance\right)}{d^{2}\times prevalence}\times \mathrm{Design}\;\mathrm{effect}{=1.96}^{2}\frac{\left(1-0.301\right)}{0.1^{2}\times 0.301}\times 1.27\;=\;1133$$

(i) N is the estimated sample size;
(ii) d is the relative precision (i.e., width of the 95% confidence interval (CI), expressed as the proportion of prevalence), aiming at a relative precision of 10% in the MRSA prevalence estimate being 10%, resulting in a margin of error of ±3.01%;
(iii) The design effect is the multiple by which the sample size might be increased compared with the sample size that would be required if simple random sampling was used.

$$\mathrm{Design}\;\mathrm{effect}=\left[1+(m+1)\frac{k^{2}\times \mathrm{prevalence}}{\left(1-\mathrm{prevalence}\right)}\right]\;=\;\left[1+(72+1)\frac{0.093^{2}\times 0.301}{\left(1-0.301\right)}\right]=1.27$$

(i) m is the estimated size of a cluster (RCHE), which is the mean number of residents from RCHEs and is estimated to be 72;
(ii) k is the coefficient of between-cluster variation, which is estimated to be 0.082 on the basis of a previous estimation [15].

Using the above equation, the estimated sample size was 1526, with approximately 22 RCHEs and 72 residents recruited. This screening activity was part of the prevention and control strategy for MDROs. A full explanation was given about the purpose of the specimen collection before taking the specimens. Verbal consent from residents or relatives was obtained for specimen collection. The specimens were obtained by trained nurses of the Infection Control Branch of Centre for Health Protection to ensure consistency. As a public health and quality improvement initiative, this program was exempt from human participant research oversight and was continuously monitored by the Centre for Health Protection, Department of Health of the HKSAR.

### 2.2. Laboratory Tests for MRSA and CRA

All the samples were sent from the RCHEs to the microbiology laboratory of Queen Mary Hospital within 45 min of transportation, and they were processed immediately. Nasal, axilla, and groin samples were cultured for the presence of MRSA and CRA using standard laboratory techniques as previously described [16,18]. Briefly, for the culture of MRSA, the clinical samples were first inoculated directly onto ChromID MRSA (bioMerieux, Marcy-l’Étoile, France) and then incubated aerobically at 35 °C for 48 h. For the culture of CRA, the clinical samples were incubated in 2 mL of brain heart infusion enrichment broth supplemented with 10 μg/mL vancomycin (Sigma-Aldrich, St. Louis, MO, USA) and 0.5 μg/mL meropenem (Hospira, Melbourne, Australia) at 35 °C for 18 h. 10 microliters of the enriched broth was subcultured onto MacConkey agar with 2 μg/mL meropenem for further incubation at 35 °C for 48 h in air. *Staphylococcus aureus* and *Acinetobacter baumannii* were identified via matrix-assisted laser desorption or ionisation time-of-flight mass spectrometry (Bruker Daltonics, Bremen, Germany). To identify the presence of methicillin resistance and carbapenem resistance, antimicrobial susceptibility tests were performed via the Kirby–Bauer disk diffusion method according to the Clinical and Laboratory Standards Institute recommendations or the manufacturer’s instructions. MRSA is defined as *Staphylococcus aureus* with in vitro resistance to cefoxitin. CRA refers to *Acinetobacter species* with in vitro resistance to imipenem. MDRA refers to any *Acinetobacter species* with in vitro resistance to the following 12 agents: amikacin, ampicillin-sulbactam, cefepime, cefoperazone-sulbactam, ceftazidime, ciprofloxacin, cotrimoxazole, gentamicin, imipenem, minocycline, piperacillin, and piperacillin-tazobactam. Internal quality control was used during culture, identification, and susceptibility testing in accordance with the accreditation by the College of American Pathologists.

### 2.3. Data Analysis

We calculated the prevalence of MRSA, CRA, and MDRA carriage among individual RCHEs by dividing the number of residents positive for these MDROs by the total number of residents surveyed in that particular RCHE. The prevalence of MDROs was further stratified by the type of home and geographical location and tested for any statistical significance. Chi-square test was used for categorical data or Fisher’s exact test (e.g., if any of the cells of a contingency table were below 5) was used, as appropriate. R software (ver. 3.6.1; https://www.r-project.org) was used for statistical analysis. For all analyses, statistical significance was defined as *p* < 0.05.

## 3. Results

**Figure 1:**
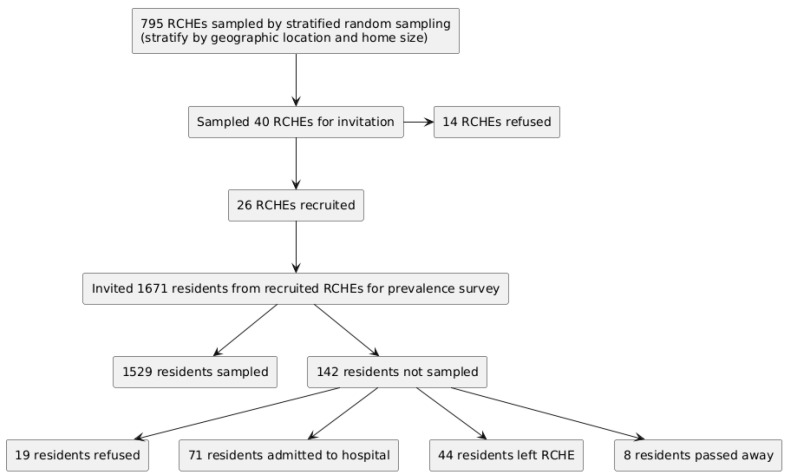
Flowchart illustrating the recruitment results of Residential Care Homes for the Elderly (RCHEs) and residents.

**Figure 2:**
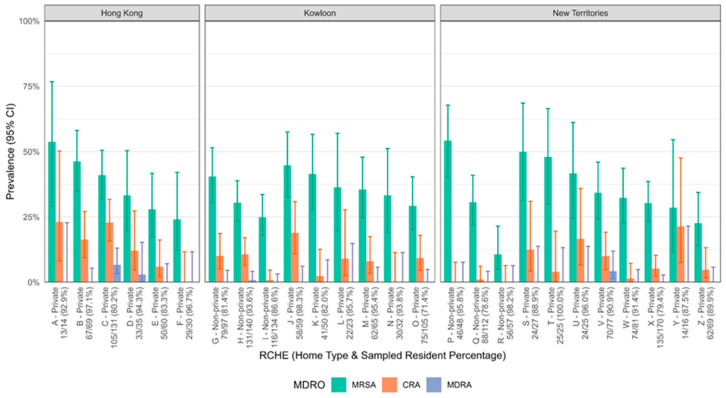
Prevalence of methicillin-resistant *Staphylococcus aureus* (MRSA), carbapenem-resistant *Acinetobacter* (CRA), and multidrug-resistant *Acinetobacter baumannii* (MDRA) in Residential Care Homes for the Elderly (RCHEs).

The recruitment results of RCHEs and residents is illustrated in Figure 1. A total of 26 RCHEs were visited from July 2022 to November 2022, and 1529 residents were recruited for sample collection. These included 20 private homes and 6 non-private homes. The size of the homes ranged from 13 to 135 residents, with a median of 57 residents. Overall, the prevalence rates of MRSA, CRA, and MDRA were 33.9% (95% CI: 31.5–36.3%), 8.1% (95% CI: 6.8–9.6%), and 0.8% (95% CI: 0.4–1.4%), respectively (Figure 2). For MRSA, the prevalence ranged from 10.7% to 51.3%. The highest rate for CRA was 21.4% (RCHE Y). In contrast, only four RCHEs (RCHE C, D, H and V) accommodated residents positive for MDRA (ranging from 0.8% to 4.3%), whereas residents of all remaining homes tested negative for MDRA.

**Figure 3:**
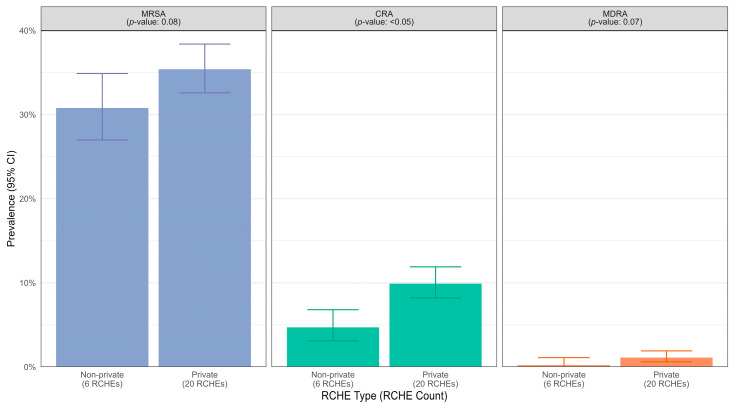
Prevalence of methicillin-resistant *Staphylococcus aureus* (MRSA), carbapenem-resistant *Acinetobacter* (CRA), and multidrug-resistant *Acinetobacter baumannii* (MDRA) in Residential Care Homes for the Elderly (RCHEs), stratified by RCHE type.

**Figure 4:**
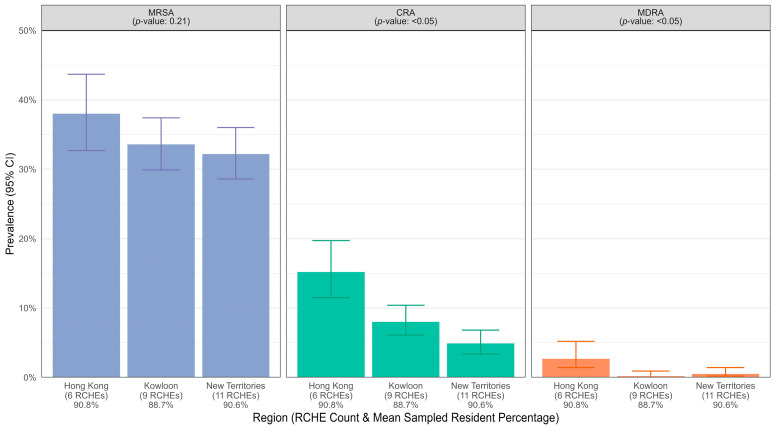
Prevalence of methicillin-resistant *Staphylococcus aureus* (MRSA), carbapenem-resistant *Acinetobacter* (CRA), and multidrug-resistant *Acinetobacter baumannii* (MDRA) in Residential Care Homes for the Elderly (RCHEs), stratified by region.

Compared with non-private homes, private homes had a greater prevalence of MDROs (Figure 3). Specifically, the prevalence of CRA was 9.9% (95% CI 8.2–11.9%) in private homes, which was higher than that of non-private homes, which was 4.7% (95% CI 3.1–6.8%), *p* < 0.05. Figure 4 shows the prevalence of MDROs stratified by geographical region. The results revealed that RCHEs in the Hong Kong region had a greater prevalence of all MDROs, followed by the Kowloon region and then the New Territories, except that the MDRA was similar for both Kowloon and the New Territories, with 0.2% (0–0.9%) and 0.5% (0.2–1.4%), respectively. The differences in the prevalence of MDROs by region were statistically significant for CRA and MDRA.

## 4. Discussion

The current study provides a territory-wide representative sample for estimating the prevalence of MDROs in RCHEs in Hong Kong. We detected a high prevalence of MRSA during the COVID-19 pandemic, with additional information on CRA that was not previously known. Such data allow us to assess the disease burden and monitor the trends of these MDROs in RCHEs. This is also crucial in the post-pandemic period as COVID-19 may have delayed impacts on antimicrobial resistance due to the extensive use of antibiotics especially in the early phase of the pandemic [19]. These estimates highlight the need for the prevention and control of these important pathogens in vulnerable groups. The results also provide valuable data for determining the economic implications and facilitate resource allocation to address the problem of antimicrobial resistance.

The reported prevalence of MRSA ranged from 6.5% to 17.2% in long-term care facilities in early years (2009–2017) [20,21,22,23,24] in the United States and European countries. In more recent years (2019–2022), a higher prevalence was detected, ranging from 28.1% to 36.0% [25,26,27]. In Hong Kong, the last territory-wide prevalence survey was conducted in 2015 with a smaller scale (20 RCHE with 1028 residents) and revealed that the prevalence of MRSA was 30.1% (95% confidence interval [CI] = 25.1–35.6%) and that of MDRA was 0.6% (95% CI = 0.1–4.1%) [15]. The current study reveals that the prevalence of MRSA is in a similar range as that reported in other developed areas (Figure 2). For both MRSA and MDRA, the prevalence rates were higher than those previously reported in the local survey. One possible reason for this increase is that resources for infection control were diverted to combat COVID-19 during the pandemic period from 2020 to 2022. For example, the isolation rooms of RCHEs were occupied by residents with COVID-19 instead of MDROs, and personal protective equipment (PPE) was limited in the initial phase of the pandemic. In addition, this point prevalence survey coincided with the fifth wave of COVID-19 in Hong Kong, during which over 1.7 million people were infected [28], including residents from RCHEs. During COVID-19 outbreaks in the RCHEs, all residents who were asymptomatic and tested negative for COVID-19 were quarantined in holding centres, which were temporarily converted from community sports centres and operated by private healthcare providers. Staff wore full PPE, including surgical respirators, face shield, gowns, and gloves while caring for these elderly individuals. Since these residents were referred from different RCHEs across Hong Kong, cross-transmission of MDROs may have occurred in the holding centres, potentially contributing to the increased prevalence of MDROs during the COVID-19 pandemic. On the other hand, the role of air dispersal in the transmission of MRSA and CRA within these institutions is worthy of further exploration [14,19].

In the subgroup analysis, we found that the prevalence of MDROs varied across different types of homes and geographical locations. In general, private RCHEs have fewer resources in terms of infrastructure and manpower and hence might have a greater chance of transmission within their facilities, resulting in a greater number of MDRO carriers. The difference in the prevalence of MDROs across geographical locations was not likely to be due to variations in the proportion of residents sampled, which had only small differences (90.8% in Hong Kong Region, 88.7% in Kowloon and 90.6% in New Territories, Figure 4). We are not aware of any organisational, structural, or implementational differences in infection and prevention procedures in RCHEs in different geographical regions that could affect the prevalence of MDROs. The Centre for Health Protection of the Department of Health has provided standardised recommendations and guidelines on the prevention and control of communicable diseases to all RCHEs in Hong Kong [29] and contact precautions for preventing transmission of MDROs within these institutions [30]. Regular training sessions are also organised to provide standardised infection control advice to all RCHE staff. Despite this, we found a higher prevalence of MRSA (38.0%) and CRA (15.2%) in the RCHEs of Hong Kong than in Kowloon and the New Territories (Figure 4). The percentage of MRSA (42.1%) identified among all *Staphylococcus aures* specimens from patients admitted to hospitals in the Hong Kong region was lower than that of Kowloon (45.2%) and comparable to that of the New Territories (42.0%) [27]. Similarly, the percentage of CRA (53.9%) identified among all *Acinetobacter* specimens from patients admitted to hospitals in the Hong Kong region was lower than that of Kowloon (62.1%) and comparable to that of the New Territories (49.3%) [31]. These results might reflect better infection control practices in hospitals in the Hong Kong region.

There are a few limitations to the present study. The average actual size of the RCHEs sampled (57) was slightly lower than the estimated one (72), implying that the prevalence of MDROs found may be biased towards smaller institutions. Nevertheless, the overall sample size was considered adequate for determining the overall prevalence of the different MDROs tested. Although the laboratory tests used were considered to be standard and should have no major problem in terms of testing sensitivity, the type of specimens and swabs used might have affected the results. For example, throat and rectal swabs combined had a higher sensitivity for MRSA than pooled keratinised skin swabs [32]. Should throat swab and rectal swabs have been added in the survey, the prevalence of MRSA detected might have been even higher. Another limitation is that no individual data, such as demographic, premorbidity, hospitalisation, and antibiotic use data, were obtained, which might be associated with the acquisition of MDROs, as shown in other studies [10,33,34]. In a systematic review involving 134 studies, old age, male sex, dementia, diabetes, cancer, chronic wounds, dependence, medical devices, previous antibiotic use, previous hospitalisation, and previous MDRO colonisation were identified as risk factors for colonisation by MDROs in long-term care facilities [35]. These factors might vary among residents with different RCHEs and result in different prevalence rates of MRSA and CRA. Including these risk factors might better explain the variation in MDRO prevalence found in different geographical areas and types (private and non-private) of RCHEs in the present study. Lastly, in the subgroup analysis, the difference in the sample size between private (20) RCHEs and non-private (6) RCHEs may limit the comparability of the results.

## 5. Conclusions

The present study revealed a continued increase in the incidence of MRSA and a high prevalence of CRA in RCHEs during the COVID-19 pandemic in Hong Kong. There is a need for continuous surveillance and stringent infection control measures for combating MDROs among this vulnerable group. In particular, we recommend targeted infection control support in private RCHEs and sustained surveillance in the post-pandemic period to further assess the AMR situation.

## Data Availability

The original contributions presented in this study are included in the article. Further inquiries can be directed to the corresponding author.

## References

[1] GBD 2021 Antimicrobial Resistance Collaborators (2024). Global burden of bacterial antimicrobial resistance 1990–2021: A systematic analysis with forecasts to 2050. Lancet.

[2] World Health Organisation Antimicrobial Resistance; Published Online November 2023. https://www.who.int/news-room/fact-sheets/detail/antimicrobial-resistance.

[3] Abubakar U., Al-Anazi M., Alanazi Z., Rodríguez-Baño J. (2023). Impact of COVID-19 pandemic on multidrug resistant gram-positive and gram-negative pathogens: A systematic review. J. Infect. Public Health.

[4] Wong S.C., Chau P.H., So S.Y., Chiu K.H., Yuen L.L., AuYeung C.H., Lam G.K., Chan V.W., Chen J.H., Chen H. (2023). Epidemiology of multidrug-resistant organisms before and during COVID-19 in Hong Kong. Infect. Prev. Pract..

[5] Ma E.S.K., Kung K.H., Chen H. (2021). Combating antimicrobial resistance during the COVID-19 pandemic. Hong Kong Med. J..

[6] Cheng V.C.-C., Wong S.-C., Ma E.S.-K., Chen H., Chiu K.H.-Y., Chen J.H.-K., So S.Y.-C., Lung D.C., Ho P.-L., Yuen K.-Y. (2025). Antimicrobial Resistance Situation and Control Measures in Hong Kong: From a One Health Perspective. J. Hosp. Infect..

[7] World Health Organisation WHO Bacterial Priority Pathogens List, 2024. https://www.who.int/publications/b/64088.

[8] World Health Organisation Health and Economic Impacts of Antimicrobial Resistance in the Western Pacific Region, 2020–2030. https://www.who.int/publications/i/item/9789290620112.

[9] You J.H.S., Choi K.W., Wong T.Y., Ip M., Ming W.K., Wong R.Y., Chan S., Tse S., Chau C.T.S., Lee N.L.S. (2017). Disease Burden, Characteristics, and Outcomes of Methicillin-Resistant *Staphylococcus aureus* Bloodstream Infection in Hong Kong. Asia Pac. J. Public Health.

[10] Cheng V.C.C., Chen J.H.K., Ng W.C., Wong J.Y.H., Chow D.M.K., Law T.C., So S.Y.C., Wong S.C.Y., Chan T.C., Chan F.H.W. (2016). Emergence of Carbapenem-Resistant Acinetobacter baumannii in Nursing Homes with High Background Rates of MRSA Colonization. Infect. Control Hosp. Epidemiol..

[11] Hübner N., Dittmann K., Begunk R., Kramer A. (2017). Infection control measures and prevalence of multidrug-resistant organisms in nonhospital care settings in northeastern Germany: Results from a one-day point prevalence study. J. Hosp. Infect..

[12] Eveillard M., LaFargue S., Guet L., Mangeol A., Piquet J., Quenon J.-L., Fauvelle F. (1999). Association between Institutionalisation and Carriage of Multiresistant Bacteria in the Elderly at the Time of Admission to a General Hospital. Eur. J. Clin. Microbiol. Infect. Dis..

[13] Ho P.L., Lai E.L., Chow K.H., Chow L.S., Yuen K.Y., Yung R.W. (2008). Molecular epidemiology of methicillin-resistant Staphylococcus aureus in residential care homes for the elderly in Hong Kong. Diagn. Microbiol. Infect. Dis..

[14] Cheng V.C., Tai J.W., Wong Z.S., Chen J.H., Pan K.B., Hai Y., Ng W.-C., Chow D.M.K., Yau M.C.Y., Cham J.W.F. (2013). Transmission of methicillin-resistant Staphylococcus aureus in the long term care facilities in Hong Kong. BMC Infect. Dis..

[15] Chen H., Au K.M., Hsu K.E., Lai C.K., Myint J., Mak Y.F., Lee S.Y., Wong T.Y., Tsang N.C. (2018). Multidrug-resistant organism carriage among residents from residential care homes for the elderly in Hong Kong: A prevalence survey with stratified cluster sampling. Hong Kong Med. J..

[16] Wong S.C., Chen J.H., Yuen L.L., Chan V.W., AuYeung C.H., Leung S.S., So S.Y.-C., Chan B.W.-K., Li X., Leung J.O.-Y. (2022). Air dispersal of meticillin-resistant *Staphylococcus aureus* in residential care homes for elderly individuals: Implications for transmission during the COVID-19 pandemic. J Hosp. Infect..

[17] Ma E.S.K. (2022). Combating antimicrobial resistance in Hong Kong: Where are we and where should we go?. Hong Kong Med. J..

[18] Wong S.C., Lam G.K., Chen J.H., Li X., Ip F.T., Yuen L.L., Chan V.W., AuYeung C.H., So S.Y., Ho P.L. (2021). Air dispersal of multidrug-resistant *Acinetobacter baumannii*: Implications for nosocomial transmission during the COVID-19 pandemic. J. Hosp. Infect..

[19] Ma E.S., Wong S.C., Cheng V.C., Wu P. (2025). Global trends and projections in antimicrobial resistance. Lancet.

[20] O’Fallon E., Schreiber R., Kandel R., D’Agata E.M. (2009). Multidrug-resistant gram-negative bacteria at a long-term care facility: Assessment of residents, healthcare workers, and inanimate surfaces. Infect. Control Hosp. Epidemiol..

[21] Jans B., Schoevaerdts D., Huang T.D., Berhin C., Latour K., Bogaerts P., Nonhoff C., Denis O., Catry B., Glupczynski Y. (2013). Epidemiology of multidrug-resistant microorganisms among nursing home residents in Belgium. PLoS ONE.

[22] Hogardt M., Proba P., Mischler D., Cuny C., Kempf V.A., Heudorf U. (2015). Current prevalence of multidrug-resistant organisms in long-term care facilities in the Rhine-Main district, Germany, 2013. Euro Surveill..

[23] Giufrè M., Ricchizzi E., Accogli M., Barbanti F., Monaco M., de Araujo F.P., Farina C., Fazii P., Mattei R., Sarti M. (2017). Colonisation by multidrug-resistant organisms in long-term care facilities in Italy: A point-prevalence study. Clin. Microbiol. Infect..

[24] March A., Aschbacher R., Sleghel F., Soelva G., Kaczor M., Migliavacca R., Piazza A., Mattioni Marchetti V., Pagani L., Scalzo K. (2017). Colonisation of residents and staff of an Italian long-term care facility and an adjacent acute care hospital geriatric unit by multidrug-resistant bacteria. New Microbiol..

[25] McKinnell J.A., Singh R.D., Miller L.G., Kleinman K., Gussin G., He J., Saavedra R., Dutciuc T.C., Estevez M., Chang J. (2019). The SHIELD Orange County Project: Multidrug-resistant Organism Prevalence in 21 Nursing Homes and Long-term Acute Care Facilities in Southern California. Clin. Infect. Dis..

[26] McKinnell J.A., Miller L.G., Singh R.D., Gussin G., Kleinman K., Mendez J., Laurner B., Catuna T.D., Heim L., Saavedra R. (2020). High Prevalence of Multidrug-Resistant Organism Colonisation in 28 Nursing Homes: An “Iceberg Effect”. J. Am. Med. Dir. Assoc..

[27] Callejón Fernández M., Madueño Alonso A., Abreu Rodríguez R., Aguirre-Jaime A., Castro Hernández M.B., Ramos-Real M.J., Pedroso-Fernandez Y., Lecuona Fernandez M. (2022). Risk factors for colonisation by carbapenemase-producing bacteria in Spanish long-term care facilities: A multicentre point-prevalence study. Antimicrob. Resist. Infect. Control.

[28] Wong S.C., Au A.K., Lo J.Y., Ho P.L., Hung I.F., To K.K., Yuen K.Y., Cheng V.C. (2022). Evolution and Control of COVID-19 Epidemic in Hong Kong. Viruses.

[29] Centre for Health Protection, Department of Health, Hong Kong Special Administrative Region Guidelines on Prevention of Communicable. Diseases in Residential Care Homes for the Elderly. https://www.chp.gov.hk/files/pdf/guidelines_on_prevention_of_communicable_diseases_in_rche_eng.pdf.

[30] Centre for Health Protection, Department of Health, Hong Kong Special Administrative Region Infection Control Advice on Multi-Drug Resistant Organisms (MDROs) for Residential Care Homes for the Elderly (RCHEs). https://www.chp.gov.hk/files/pdf/infection_control_advice_on_mdro_for_rche_eng.pdf.

[31] Centre for Health Protection, Department of Health, Hong Kong Special Administrative Region Statistics on Antimicrobial Resistance Control. https://www.chp.gov.hk/en/statistics/data/10/100044/6864.html.

[32] Batra R., Eziefula A.C., Wyncoll D., Edgeworth J. (2008). Throat and rectal swabs may have an important role in MRSA screening of critically ill patients. Intensive Care Med..

[33] Lim C.J., Cheng A., Kennon J., Spelman D., Hale D., Melican G., Sidjabat H.E., Paterson D.L., Kong D.C.M., Peleg A.Y. (2014). Prevalence of multidrug-resistant organisms and risk factors for carriage in long-term care facilities: A nested case-control study. J. Antimicrob. Chemother..

[34] Denis O., Jans B., Deplano A., Nonhoff C., De Ryck R., Suetens C., Struelens M.J. (2009). Epidemiology of methicillin-resistant Staphylococcus aureus (MRSA) among residents of nursing homes in Belgium. J. Antimicrob. Chemother..

[35] Rodríguez-Villodres Á., Martín-Gandul C., Peñalva G., Guisado-Gil A.B., Crespo-Rivas J.C., Pachón-Ibáñez M.E., Lepe J.A., Cisneros J.M. (2021). Prevalence and Risk Factors for Multidrug-Resistant Organisms Colonisation in Long-Term Care Facilities Around the World: A Review. Antibiotics.

